# High seroprevalence of antibodies against SARS-CoV-2 among healthcare workers 8 months after the first wave in Aden, Yemen

**DOI:** 10.1371/journal.pgph.0000767

**Published:** 2022-11-09

**Authors:** Rami Malaeb, Nagwan Yousef, Omar Al-Nagdah, Qassem Hussein Ali, Mohammed Ali Saleh Saeed, Amna Haider, Evgenia Zelikova, Nada Malou, Sonia Guiramand, Clair Mills, Francisco Luquero, Klaudia Porten

**Affiliations:** 1 Epicentre, Dubai, United Arab Emirates; 2 Médecins Sans Frontières, Aden, Yemen; 3 Médecins Sans Frontières, Paris, France; 4 Epicentre, Paris, France; PLOS: Public Library of Science, UNITED STATES

## Abstract

The true burden of COVID-19 in Yemen is underestimated. The healthcare system is dysfunctional and there is a high shortage of health care workers in the country. Testing for SARS-CoV-2 remains limited and official surveillance data is restricted to those who are severe or highly suspected. In this study, Médecins Sans Frontières (MSF) aimed to conduct serological screening using rapid tests for asymptomatic staff at the MSF Aden Trauma Center to determine the SARS-CoV-2 antibody seropositivity. Four months after the peak of the first wave, we offered all the staff at the MSF Aden Trauma Center PCR if symptomatic, and a baseline SARS-CoV-2 serology screening followed by follow-up screenings. A final round was scheduled four months after the baseline. A rapid serology lateral flow test, NG-Test IgM-IgG was used in all rounds and in the final round, an electrochemiluminescence immunoassay (ECLIA) (Elecsys Anti-SARS-CoV-2 assay). Univariate and multivariate analyses were used to identify risk factors for seropositivity. The level of agreement between the different serology assays used was investigated. Overall 69 out of 356 participants (19.4%, 95% CI 17.9–20.8) tested positive by NG-Test between September and November 2020. A sub-sample of 161 staff members were retested in January 2021. Of these, the NG-Test detected only 13 positive cases, whereas the ECLIA detected 109 positive cases. The adjusted seroprevalence by ECLIA was 59% (95%CI 52.2–65.9). The non-medical staff had significantly lower odds of seropositivity compared to the medical staff (AOR 0.43, 95% CI 0.15–0.7, p<0.001). The positive percent agreement between the two tests was very low (11%). Our results suggest a very high SARS-CoV-2 seroprevalence in healthcare workers in Yemen, highlighting the need for regular testing and rapid vaccination of all healthcare workers in the country.

## Introduction

After seven years of civil war, Yemen is facing the worst humanitarian crisis in the world. Around 80 percent of the Yemeni population (24.1 million people) are in need of humanitarian assistance [1, 2]. The health care system is crippled, with more than half of the country’s health facilities dysfunctional and a severe shortage of skilled medical personnel [1, 3]. Since 2017, Yemen has faced the largest cholera outbreak in the world besides other epidemics of diphtheria, dengue, and malaria, which thrived as a consequence of the conflict [2]. Adding to this dire situation, Yemen was challenged with the COVID-19 pandemic in 2020 [4].

The true burden of COVID-19 in Yemen remains unknown. As of 18 May 2021, the total number of official cases reported was 6,572 and 1,295 associated deaths. The first wave of the COVID-19 outbreak in Yemen was between May and September 2020, with the highest weekly number of cases (244 cases) recorded in epi week 24 (June 8 to June 14, 2020). Very few cases were reported between October 2020 and January 2021. In February 2021, the number of weekly cases started to increase significantly, indicating the start of the second wave in the country. During epi week 13 of 2021, the country recorded its highest weekly number of reported cases (765 confirmed cases and 95 deaths [5]). The naïve case fatality rate (CFR) of 19.7% was the highest reported worldwide, but was largely due to the limited number of tests performed, poor access to testing, and under-reporting of COVID-19 cases (with only highly suspected cases tested [4, 6]) and deaths [3].

Protecting health care workers (HCWs) in Yemen is critical as the country has already fewer than 10 health workers for every 10,000 people. Médecins Sans Frontières (MSF) prioritized the protection of its staff in Yemen by ensuring the availability of personal protective equipment (PPE) and by strengthening Infection Prevention and Control (IPC) measures in its facilities [7]. Despite this, staff absence due to illness was high during the first peak. MSF needed to rapidly identify, and manage staff illness, and also understand better community transmission in Aden, given the limited information available from official testing.

In this study we aimed to conduct serological screening using rapid tests for asymptomatic staff at the MSF Aden Trauma Center to determine the SARS-CoV-2 antibody seropositivity. When the study was conceived in mid-2020, access to reverse transcription polymerase chain reaction (RT-PCR) test was extremely limited and validated rapid antigen tests were not available. Therefore, we performed serological screening using rapid tests for asymptomatic staff, reserving PCR for those with symptoms, as this was the only feasible approach. An electrochemiluminescence immunoassay (ECLIA) became available in November 2020 in Aden and was used in the final round of screening, given its superior performance. Therefore the main objectives of this study were to measure the seroprevalence of antibodies to SARS-CoV-2 in the MSF staff at the Aden Trauma Center, to identify the potential risk factors for seropositivity, and to compare the results of the serology lateral flow test with the electrochemiluminescence immunoassay used in the final screening.

## Methods

### Enrolment of study subjects

All staff members (n = 404) were invited to take part in the study on a voluntary basis. A written consent was obtained from all the study participants. The screening appointments were organized during information sessions provided by the study team. On the day of the screening, participants received an awareness session on COVID-19 prevention, an explanation of the study objectives, and interpretation of their serological test result.

### Data collection

A baseline SARS-CoV-2 serology screening was conducted between September 27^th^ and October 4^th^, 2020. Then three follow up screenings took place every two weeks, between October 11^th^ and November 12^th^, 2020. In the follow-up rounds, new participants and those who tested negative in previous screenings were targeted. A final screening was scheduled on the 10^th^ of January 2021 after access to ECLIA testing became possible. As it was not feasible to invite all the staff members to participate in the final round, a sub-sample was invited including 61 of those who had tested positive in previous rounds, and 100 among those who had tested negative. The final round aimed to measure the change in the results of seropositivity in the lateral flow immunoassay (NG-Test) over the study period and to assess the test agreement between the two different assays used.

For each participant, we collected baseline information on sex, age, job role, existing chronic illnesses, and self-reported adherence to COVID-19 related IPC measures. In each testing round, the study doctor or nurse performed an assessment of any current symptoms associated with COVID-19 and recent potential exposure to SARS-CoV-2. Participants who met the WHO case definition of a suspect COVID-19 case were not tested for antibodies but were offered diagnostic testing using RT-PCR.

### Tests for presence of antibodies to SARS-CoV-2

We used a rapid serology lateral flow test, NG-Test IgM-IgG COVID-19 (NG Biotech, Guipry, France) according to the manufacturer’s instructions. The NG-Test detects specific IgG and IgM antibodies using the nucleocapsid protein (N) as the target antigen. As reported by the manufacturer, the test has 100% sensitivity and specificity (CI95% [56.1–100]) and (CI95% [85–100]), respectively, at 15 days after the onset of symptoms. The NG-Test was used in all the screening rounds of our study [8].

In the final screening, we also used an electrochemiluminescence immunoassay (ECLIA) by Elecsys Anti-SARS-CoV-2 assay using the cobas e411 module (Roche Diagnostics, Rotkeuz, Switzerland). The test provides a qualitative detection of all antibodies (including IgG) to SARS-CoV-2 in human serum and plasma [9]. The test result is expressed as cut-off index (COI) value, calculated by dividing the electrochemiluminescence signal of the sample with the signal obtained by calibration. The cut-off is 1.0 COI [10]. As reported by the manufacturer, the test has a sensitivity of 99.5% (CI95% [97–100]) and specificity of 99.8% (CI95% [99.7–99.9]) [9]. Serum samples were collected by the MSF laboratory team and analyzed at the laboratory of the Aden National Blood Transfusion and Research Center in agreement with MSF and Epicentre.

#### Statistical analysis

We described the study participants using summary measures, and estimated seroprevalence with 95% confidence intervals (CI95%), by type of job, IPC adherence and recent exposure, in addition to the baseline characteristics. We performed univariate logistic regression analysis and constructed a multivariate logistic regression model including all variables that could potentially explain the differences in seropositivity. The finite population correction (fpc) factor was included in the calculation of our estimated parameters. Weighted estimates were calculated in the final screening results to adjust for the oversampling of the cases with positive rapid test results. Rao-Scott Chi-square tests were used to compare categorical variables and a p-value of less than 0.05 was considered statistically significant. In the absence of a reference standard test to measure the sensitivity and specificity of the diagnostic tests used, we calculated the positive percent agreement (NPA) and the negative percent agreement (PPA), in addition to the Kappa measure of agreement to compare the results of the NG-Test and Elecsys used in the final screening. For all analyses, we used RStudio v.1.2.5033 statistical software.

#### Ethical considerations

This protocol was approved by the MSF Ethics Review Board on July 30, 2020, reference number 2056a, and by Aden University Institutional Review Board on August 9, 2020 (Research Code: REC-79-2020).

## Results

### Time period screening implementation

**Fig 1:**
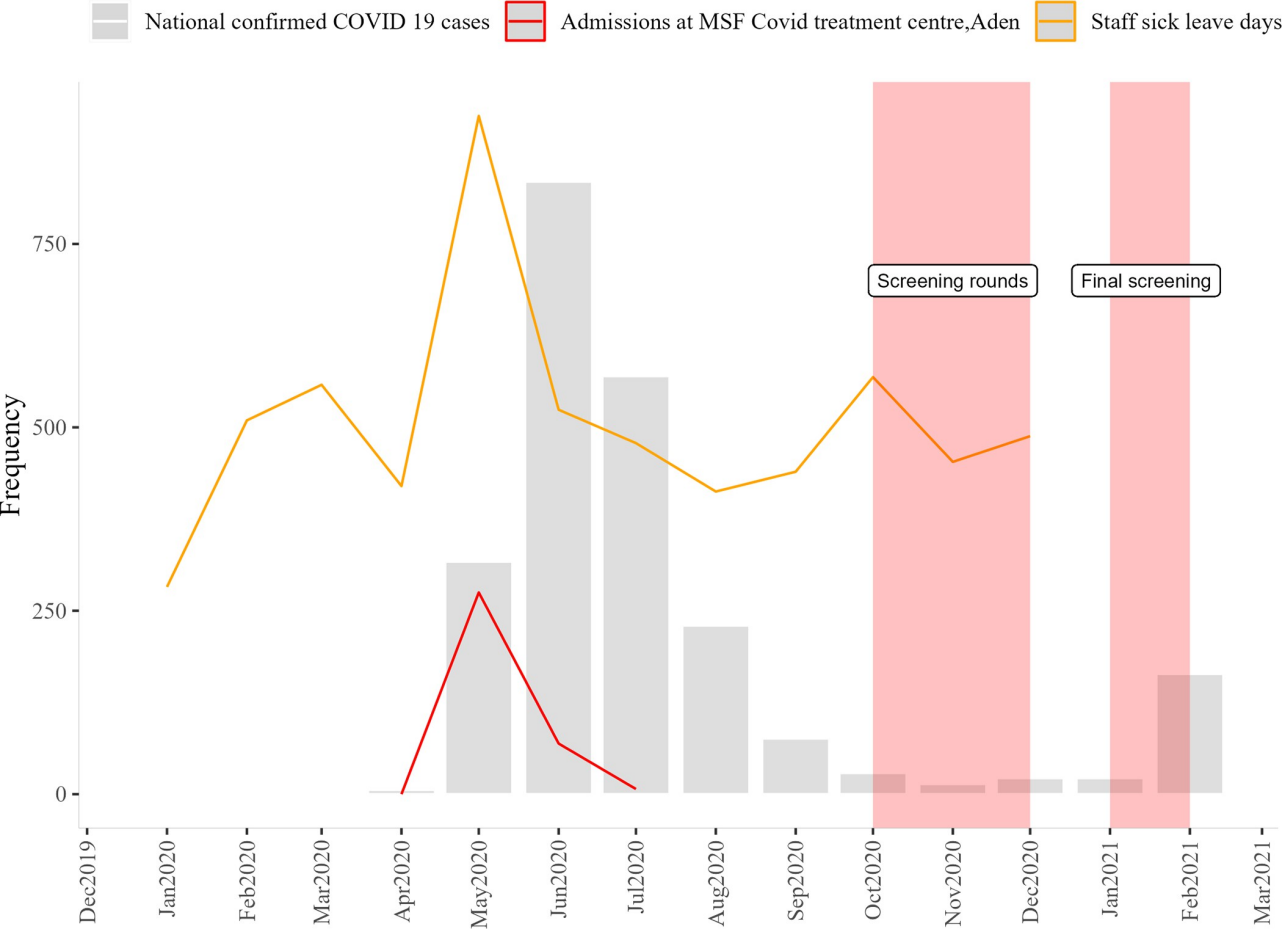
Yemen COVID-19 epi curve, number of admissions at MSF covid19 treatment center and number of sick leave days taken by MSF staff in Aden Trauma Center—Sep 2020 and Jan 2021.

The baseline screening took place at the end of September 2020, which was four months after the peak of the outbreak in June 2020 (Fig 1). During the study period (Sep 2020- Jan 2021) the number of official cases reported in the country was low, ranging from 1 to 10 cases per week, suggesting low transmission. The last round was performed 8 months after the first peak.

### Characteristics of study participants

A total of 356/404 staff members working at the MSF Aden Trauma center agreed to participate in the study. Participants were predominantly male (87%) with a mean age of 37.8 years (SD = 8.4). The majority reported no chronic illnesses (80%). Most participants had a permanent work contract (79%); the remainder were daily or temporary workers. Forty-nine percent were medical staff, including physicians, nurses, and physical therapists. Twenty five percent of participants were other health personnel who had indirect patient contact, such as laboratory technicians, IPC officers, and cleaners. The other participants (26%) worked at the health facility with no link to patient care such as watchmen, drivers, and administration staff. Over half attended at least one IPC training session related to COVID-19 (51%). Forty-eight percent indicated being a contact of a suspect or confirmed COVID-19 case during the two weeks prior to their screening. During the study period, 19 percent reported COVID-19 related symptoms and only one tested PCR positive during the study period.

### Overall estimate of infection based on seropositivity

The total number of participants who tested positive by NG-Test IgG-IgM in any of the rounds between the initial screening and end of follow-up was 69/356. Hence, the overall SARS-CoV-2 seropositivity measured by rapid test was 19.4% (95% CI 18–20.7). The IgG and IgM seropositivity were 17% (CI95% [15.8–18.4]) and 18.5% (CI95% [17.2–19.9]), respectively. Characteristics of those who were seropositive by NG-Test are summarized in Table 1. (The results of each round are presented in Table A in S1 File).

**Table 1:** SARS-CoV-2 seroprevalence results detected by NG-Test in MSF Aden Trauma Center during follow-up screenings from 27 Sep to Nov 8 2020 and a final screening from 10 to 24 Jan 2021.

Characteristics	NG-Test Results*		NG-Test Results*	
	Sep 27 to Nov 8 2020		10 to 24 Jan 2021	
	N	Prevalence [CI 95%]	N	Prevalence [CI 95%]
**Overall**	69/356	19.4% [18–20.7]	13/161	8% [6.6–9.4]
**Sex**				
*male*	64/310	20.6% [19.11–22.09]	12/144	8.3% [6.79–9.8]
*female*	5/46	10.8% [7.8–13.8]	1/17	5.9% [2.2–9.6]
**Age Group (years)**				
*20–29*	8/44	18.18% [14.5–21.9]	1/19	5.3% [2–8.6]
*30–39*	30/183	16.4% [14.6–18.2]	5/76	6.57% [4.7–8.4]
*40–49*	21/92	22.82% [20.1–25.6]	4/47	8.5% [5.8–11.2]
*50–59*	7/28	25% [19.7–30.3]	1/13	7.7% [2.9–12.5]
*60+*	3/9	33.3% [23.1–43.5]	2/6	33.3% [20.7–45.9]
**Type of contract**				
*daily work*	12/45	26.6% [22.3–30.9]	1/20	5% [0.9–9.1]
*temporary*	2/29	6.9% [3.8–10]	0/9	0% [NA]
*permanent*	55/282	19.5% [18 – 21]	12/132	9% [7 – 11]
**Job role**				
*medical Staff*	45/173	26% [23.8–28.2]	8/82	9.7% [7.5–11.9]
*other personnel at health facility*	11/90	12.2% [10–14.4]	3/42	7.14% [4.4–9.8]
*other health personnel*	13/93	14% [11.7–16.4]	2/37	5.4% [2.9–7.9]
**Comorbidities**				
*yes*	16/71	22.5% [19.3–25.7]	2/41	4.9% [2.6–7.2]
*no*	53/285	18.6% [17–20.1]	11/120	9.2% [7.4–11]
**Attended IPC Training**				
*yes*	30/181	16.6% [14.8–18.4]	6/82	7.3% [5.3–9.3]
*no*	39/175	22.3% [20.3–24.3]	7/79	9% [6.7–11]
**PPE use at work**				
*always*	69/349	19.7% [18.3–21]	13/158	8.2% [NA]
*not always*	0/7	0	0/3	0
**Contact with suspect/confirmed case in the past 2 weeks**				
yes	25/169	14.8% [13–16.6]	9/81	11% [8.8–13.5]
no	44/186	23.6% [21.6–25.6]	4/80	5% [3.4–6.6]

*estimates adjusted using finite-population correction factor (fpc).

In January 2021, 161 participants, of whom 61 were positive by NG-Test in previous rounds, were re-tested. 13 (8%, 95% CI 6.6–9.5) were seropositive by NG-Test whereas 109 (67.7%, CI95%[65.3–70.1]) were positive by ECLIA. Adjusting for oversampling of cases with positive NG-Test results, the weighted seroprevalence by ECLIA was 59% (95% CI 52.2–65.9).

#### Univariate and multivariate risk factor analyses of seropositivity

Univariate analysis based on the 161 participants with ECLIA results shows that daily workers were significantly more likely than permanent staff to be infected by SARS-CoV-2 (OR = 1.44, p = 0.05), however, temporary staff had less the odds of being infected compared to permanent staff (OR = 0.6, p = 0.04) (Table 2). The non-medical staff were 38% less likely to be seropositive compared to the medical personnel (OR = 0.38, p < 0.001). Staff members who reported comorbidities were less likely to be infected by SARS-CoV-2 (OR = 0.77, p = 0.043). Those who reported being a contact with a suspect COVID-19 case had lower the odds of seropositivity compared to those who reported no exposure (OR = 0.69, p = 0.001). Multiple regression analyses retain significant associations only for non-medical personnel and being a contact (OR = 0.43, p < 0.01 & OR = 0.72, p = 0.008, respectively) (see Table B in S1 File). Analyses based on NG-Test results are presented in Table C in S1 File.

**Table 2:** Univariable analyses for risk factors of seropositivity to SARS-CoV-2 in 161 participants in the MSF Aden Trauma Center.

	N	Crude Prevalence [CI 95%]	Weighted* Prevalence [CI 95%]	Univariate OR [CI 95%]	P-value
**Overall**	109/161	67.7% [65.3–70.1]	59% [52.2–66]	-	-
**Sex**					
*male*	97/144	67.4% [64.8–67]	58.4% [51.1–65.6]	Ref	-
*female*	12/17	70.6% [63.3–77.8]	64.5% [45–84]	1.16 [0.8–1.51]	0.42
**Age group (years)**					
*20–29*	13/19	68.4% [61.3–74.5]	60% [40.4–76.6]	Ref	-
*30–39*	51/76	67.1% [63.6–70.6]	59.3% [49.3–69.3]	0.94 [0.59–1.29]	0.74
*40–49*	32/47	68% [63.7–72.3]	60% [47–73]	0.98 [0.6–1.35]	0.93
*50–59*	10/13	76.9% [69.3–84.5]	66.5% [41–92]	1.53 [1–2.06]	0.11
*60+*	3/6	50% [36.7–63.3]	30% [20 – 59]	0.46 [-0.17–1.09]	0.01
**Type of contract**					
*daily work*	15/20	75% [68.7–81.3]	66% [46.4–85.6]	1.44 [1.06–1.81]	0.05
*temporary*	5/9	55.5% [44.7–66.3]	52.5% [23.1–82]	0.6 [0.13–1.07]	0.04
*permanent*	89/132	67.4% [64.7–70.1]	58.6% [51–66.2]	Ref	-
**Job role**					
*medical Staff*	60/82	73.2% [70–76.3]	63.6% [53.8–73.4]	Ref	-
*other health personnel*	30/42	71.4% [66.9–75.9]	66.3% [53.2–79.4]	0.9 [0.62–1.17]	0.54
*other personnel at health facility*	19/37	51.3% [46–55.6]	42.3% [28.2–56.4]	0.38 [0.12–0.63]	<0.01
**Comorbidities**					
*no*	53/285	18.6% [17–20.1]	61% [47.3–74.7]	Ref	-
*yes*	16/71	22.5% [19.3–25.7]	53.6 [45.8–61.4]	0.77 [0.53–1]	0.043
**Attended IPC training**					
*no*	54/79	68.3% [65–71.7]	60% [50–70]	Ref	-
*yes*	55/82	67% [63.7–70.3]	60% [50–69.6]	0.94 [0.72–1.15]	0.6
**PPE use at work**					
*not always*	2/3	66.7% [49–87.3]	66.6% [21.5–112]	Ref	-
*always*	107/158	67.7% [65.4–70]	59% [52.1–65.9]	1.04 [0.24–1.84]	0.9
**Contact with suspect/confirmed case in the past 2 weeks**					
no	58/81	71.6% [64–81.3]	61.6% [51.8–71.4]	Ref	-
yes	51/80	63.7% [60.2–67.2]	57% [47.1–66.7]	0.69 [0.47–0.9]	0.001

*Weighted by NG-Test results of previous rounds to adjust for over-sampling of RDT positive.

### Test agreement between Elecsys and NG-Test

**Fig 2:**
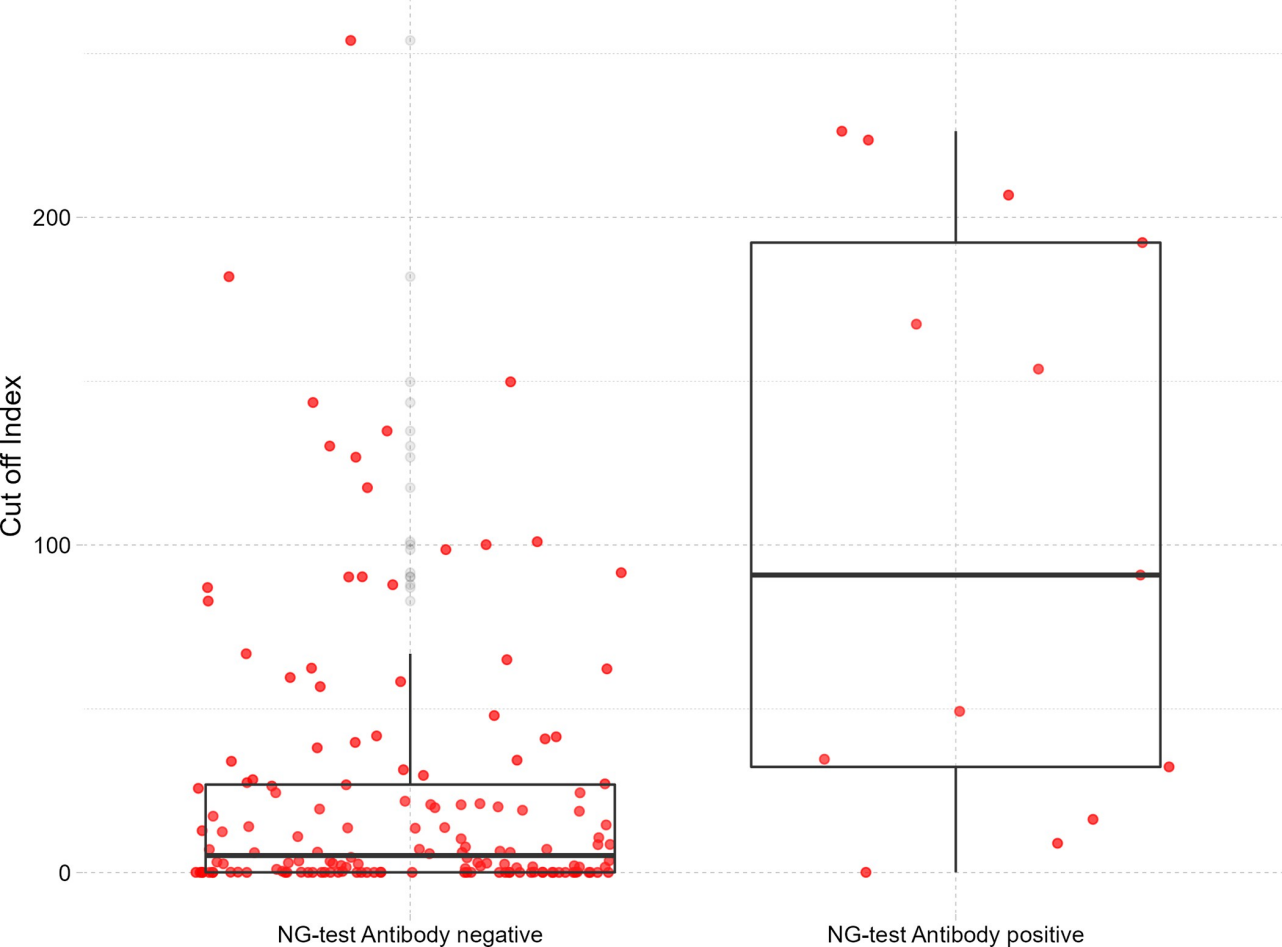
Boxplot of Elecsys COI levels by NG-Test results of the initial and follow-up screenings (61 positive & 100 negative) in MSF Aden Trauma Center–January 2021.

The test agreement was assessed in the last round of 161 samples that were tested by both the NG-Test and Elecsys immunoassay. As shown in Table 3, the positive percent agreement (PPA) of these two testing methods was 11% (95% CI [5.8–18.4]) and the negative percent agreement (NPA) was 98% (95% CI [89.7–99.9]), with a kappa coefficient of 0.061 (95% CI [0.012–0.11]) indicating slight agreement. In our analysis, the cut-off index (COI) values generated by Elecsys ranged between 0.059 and 254 with a mean of 30.8 and a median of 6.54 (IQR [0.096–33.55]). The majority of COI results were below 30. The median COI among those who tested negative by NG-Test in previous rounds was 0.36 (95%CI [0.15–1.52]) compared to 45.48 (95%CI [40.7–58.4]) among those who tested positive (p <0.001) (Fig 2).

**Table 3:** Test agreement between NG-Test and Elecsys in 161 samples in MSF Aden Trauma Center–January 2021.

		NG-Test		
		Positive, n	Negative, n	Total, N
Elecsys	Positive, n	12	97	109
	Negative, n	1	51	52
	Total, N	13	148	161

Note: Positive Percent Agreement (PPA) = 12 / 109 = 0.11 | Negative Percent Agreement (NPA) = 51/52 = 0.98.

## Discussion

To our knowledge, this study is the first to report on the prevalence of SARS-CoV-2 antibodies among HCWs in Yemen. Our results show that seropositivity in this cohort was high (19.4%) using rapid serology lateral flow tests. In the last screening round, the ECLIA, which has superior performance, suggested that the true extent of infection was grossly underestimated, as it was over eight times higher than what was detected by the NG-Test, with an overall adjusted seroprevalence estimate of 59%.

A systematic review and meta-analysis estimated an overall seroprevalence of 8.7% (range 0%-45.3%) among HCWs globally [11]. The adjusted seroprevalence of 59% in our study may be one of the highest to be reported in HCWs in a non-COVID-19 hospital worldwide. Similar studies conducted in other neighboring countries have shown much lower seropositivity in HCWs; for example, in a tertiary hospital in Saudi Arabia a seroprevalence of 6.3% was reported [12]. Another seroprevalence study using ELISA and ECLIA in a COVID-19 hospital in Oman reported an infection rate of 10.5% [13]. This highlights the wide gap between the conflict setting of Aden and other countries demonstrating the direct impact of the war on the healthcare system and its diminished capacity to protect its HCWs.

Several risk factors associated with a positive SARS-CoV-2 antibody test in HCWs have been reported [11]. A higher probability of SARS-CoV-2 seropositivity was observed in HCWs working in COVID-19 units. Similarly, front-line HCW providing patient care have been reported to have higher odds of seropositivity [11, 14]. Our results concur with these findings, as medical and nursing staff were at higher risk of SARS-CoV-2 infection compared to other staff. The fact that daily workers had higher rates of infection than those on temporary or permanent contracts suggests that either they were exposed to very high levels of community exposure outside the workplace, or perhaps were less likely to adhere to protective measures at work. The majority of staff reported using PPE at work; this also suggests that community exposure to SARS-CoV-2 infection was important, although correct PPE and IPC measures may not always have been respected in the workplace.

Unlike other studies that showed a significant association of higher seropositivity in contacts of a confirmed or a suspect case [11], in our study cohort those who reported being a contact had lower odds of being seropositive. This was not expected, but transmission was very low in the community during the study period, as reported by the national surveillance; hence participants could have over-reported being in contact with “suspect COVID-19” cases, which were rather due to illness from other causes besides that the wide definition of a suspect case that could have led to an overestimation in the reporting.

Several studies have shown that the use of serological rapid diagnostic tests in low-resource settings has limitations and they are not optimal for active surveillance [15–18]. The NG-Test may have lower specificity for IgM and results for IgM should be interpreted with caution [19, 20]. Rapid serological tests have not been robustly evaluated for use in time periods late (more than several months) after infection. The use of Elecsys in the final screening gave us the opportunity to increase the sensitivity in identifying participants with SARS-CoV-2 antibodies. This showed that the NG-Test missed many Elecsys positive cases, leading to a high proportion of false negatives (97 out of 109) and very low level of agreement in the results of the final round (8% vs 67.7% seropositivity). Studies evaluating Elecsys have consistently reported high performance and reliability [21–23].

Information on onset of symptoms in our study cohort was lacking, but there is strong epidemiological evidence suggesting that the majority of infections among the staff occurred during the first peak of transmission. This is reinforced by the high level of staff absence due to illness observed in this period. This could also partly explain the poor performance of NG-Test in our study context; more than 4 months after infection, antibody levels may have been below the level of detection of this test. Additionally, the Elecsys COI levels were lower among those who tested negative by NG-Test. Though COI levels do not directly quantify the antibody titer, other studies have shown that it is closely associated with the assay composition and reaction kinetics [10]. Other studies have also shown that the antibody titer level is associated with the severity of infection [15]. There were no recorded hospitalizations for COVID-19 among the study participants; we thus assume that the COVID-19 infections that occurred in our study participants were asymptomatic or mild.

Our study has limitations. Participation in the study was voluntary; however the majority (over 88%) of staff agreed to participate in the study, limiting potential bias. Information and recall bias could have affected the measurement of potential risk factors including reporting of the use of PPE, attending IPC training and being a contact. The unavailability of ECLIA or other confirmatory tests was also a constraint, unavoidable in this context, and only a sub-population could be screened with both tests. The limitations and performance of the serology tests could have also resulted in biased estimates; however, the use of the ECLIA in the last round confirms a high infection rate and low false positive rapid test results.

In conclusion, the results presented in our study highlight a very high SARS-CoV-2 seroprevalence in HCWs in Yemen. In a country with a fragile health system and underreporting of cases, this study confirms evidence from other sources of extremely high transmission of COVID-19 in Aden in 2020 [23], including a high seroprevalence rate of 27.4% that was estimated in a population-based survey conducted during the same period of our study [24]. Rapid vaccination of all health workers and reinforcement of IPC and barrier measures is key to preventing morbidity and potentially preventable deaths, especially hospital acquired infections in staff and patients with risk factors for severe disease.

## Supporting information

S1 File Tables A-C. (DOCX)

S1 Questionnaire Inclusivity in global research. (DOCX)

## References

[1] Yemen and COVID-19: The pandemic exacts its devastating toll n.d. https://www.brookings.edu/blog/future-development/2020/06/15/yemen-and-covid-19-the-pandemic-exacts-its-devastating-toll/ (accessed April 17, 2021).

[2] Coronavirus will “delete Yemen from maps all over the world” | World News | Sky News n.d. https://news.sky.com/story/coronavirus-will-delete-yemen-from-maps-all-over-the-world-11989917 (accessed April 17, 2021).

[3] Fallon K, Moran A, Dunia A. A tipping point for Yemen’s Health System: The Impact of COVID-19 in a Fragile State. https://medglobal.org/wp-content/uploads/2020/07/A-Tipping-Point-for-Yemen%E2%80%99s-Health-System072020.pdf (accessed April 17, 2021).

[4] Ali Maher O, Pichierri G, Farina G, Panu Napodano CM, Bellizzi S. COVID-19 Response and Complex Emergencies: The Case of Yemen. Disaster Med Public Health Prep 2020;14:E27–8. doi: 10.1017/dmp.2020.271 32713416 PMC7443562

[5] Yemen: WHO Coronavirus Disease (COVID-19) Dashboard With Vaccination Data | WHO Coronavirus (COVID-19) Dashboard With Vaccination Data n.d. https://covid19.who.int/region/emro/country/ye (accessed April 17, 2021).

[6] Dhabaan GN, Al-Soneidar WA, Al-Hebshi NN. Challenges to testing COVID-19 in conflict zones: Yemen as an example. J Glob Health 2020;10:1–3. doi: 10.7189/jogh.10.010375 32582438 PMC7307803

[7] MSF’s activities on coronavirus COVID-19 | MSF n.d. https://www.msf.org/covid-19-depth (accessed April 17, 2021).

[8] Dortet L, Emeraud C, Vauloup-Fellous C, Khecharem M, Ronat JB, Fortineau N, et al. Rapid Determination of SARS-CoV-2 antibodies using a bedside, point-of-Care, serological test. Emerg Microbes Infect 2020;9:2212–21. doi: 10.1080/22221751.2020.1826892 32969769 PMC7580567

[9] Elecsys® Anti-SARS-CoV-2 n.d. https://diagnostics.roche.com/global/en/products/params/elecsys-anti-sars-cov-2.html (accessed April 13, 2021).

[10] Lapić I, Rogić D, Šegulja D, Kralik Oguić S, Knežević J. The reliability of SARS-CoV-2 IgG antibody testing—a pilot study in asymptomatic health care workers in a Croatian university hospital. Croat Med J 2020;61:485–90. doi: 10.3325/cmj.2020.61.485 33410294 PMC7821371

[11] Galanis P., Vraka I., Fragkou D., Bilali A., Kaitelidou D. Seroprevalence of SARS-CoV-2 antibodies and associated factors in healthcare workers: a systematic review and meta-analysis. Journal of Hospital Infection 2021;108:120–34. doi: 10.1016/j.jhin.2020.11.008 33212126 PMC7668234

[12] Ahmed WA, Dada A, Alshukairi AN, Sohrab SS, Faizo AA, Tolah AM, et al. Seroprevalence of Neutralizing Antibodies to Severe Acute Respiratory Syndrome Coronavirus 2 (SARS-CoV-2) among Healthcare Workers in Makkah, Saudi Arabia. J King Saud Univ—Sci 2021;33:101366. doi: 10.1016/j.jksus.2021.101366 33613011 PMC7881290

[13] Al-siyabi N, Al-lawati A, Al-lawati M, Al-wahibi I, Al-M. Characteristics and Immunoglobulin G Seropositivity among Covid-19 Positive Healthcare Workers in a Tertiary Care Hospital in Oman 2021:0–15. 10.5001/omj.2021.116. PMC8503324 34676110

[14] Mostafa A, Kandil S, El-Sayed MH, Girgis S, Hafez H, Yosef M, et al. SARS-CoV-2 seroconversion among 4040 Egyptian healthcare workers in 12 resource-limited healthcare facilities: A prospective cohort study. Int J Infect Dis 2021;104:534–42. doi: 10.1016/j.ijid.2021.01.037 33484863 PMC7817419

[15] Eyre DW, Lumley SF, Donnell DO, Stoesser NE, Matthews PC, Howarth A, et al. Stringent thresholds in SARS-CoV-2 IgG assays lead to under-detection of mild infections. BMC Infect Dis 2021;21:187. doi: 10.1186/s12879-021-05878-2 33602152 PMC7889711

[16] Russo A, Calò F, Di Fraia A, Starace M, Minichini C, Gentile V, et al. Assessment and comparison of two serological approaches for the surveillance of health workers exposed to SARS-CoV-2. Infect Drug Resist 2020;13:4501–7. doi: 10.2147/IDR.S282652 33364797 PMC7751610

[17] Mukwege D, Byabene AK, Akonkwa EM, Dauby N, Buhendwa JC, Coadou A Le, et al. High SARS-CoV-2 Seroprevalence in Healthcare Workers in Bukavu, Eastern Democratic Republic of Congo 2021:1–5. doi: 10.4269/ajtmh.20-1526 33591936 PMC8045652

[18] Pallett SJC, Rayment M, Patel A, Fitzgerald-Smith SAM, Denny SJ, Charani E, et al. Point-of-care serological assays for delayed SARS-CoV-2 case identification among health-care workers in the UK: a prospective multicentre cohort study. Lancet Respir Med 2020;8:885–94. doi: 10.1016/S2213-2600(20)30315-5 32717210 PMC7380925

[19] Garlantézec R, Heslan C, Tadie E, Tattevin P, Thibault V, Paris C. A lateral flow immunoassay test performance in SARS-CoV-2 seroprevalence surveys: a validation study among healthcare workers. Emerg Microbes Infect 2020;9:2547–9. doi: 10.1080/22221751.2020.1852893 33206004 PMC7717860

[20] Kobashi Y, Shimazu Y, Nishikawa Y, Kawamura T, Kodama T, Obara D, et al. The difference between IgM and IgG antibody prevalence in different serological assays for COVID-19; lessons from the examination of healthcare workers. Int Immunopharmacol 2021;92. doi: 10.1016/j.intimp.2020.107360 33508702 PMC7836839

[21] Afzal N, Tariq N, Raza S, Shakeel D. Diagnostic Accuracy of Electro-Chemiluminescence Immunoassay Anti-SARS-CoV-2 Serological Test. Cureus 2021;13:1–6. doi: 10.7759/cureus.12588 33575149 PMC7870122

[22] Egger M, Bundschuh C, Wiesinger K, Gabriel C, Clodi M, Mueller T, et al. Comparison of the Elecsys® Anti-SARS-CoV-2 immunoassay with the EDI^TM^ enzyme linked immunosorbent assays for the detection of SARS-CoV-2 antibodies in human plasma 2020. Clinica Chimica Acta 2020;509:18–21. 10.1016/j.cca.2020.05.049. PMC7261064 32485155

[23] Koum Besson E, Norris A, Ghouth ASB, Freemantle T, Alhaffar M, Vazquez Y, et al. Excess mortality during the COVID-19 pandemic: A geospatial and statistical analysis in Aden governorate, Yemen. BMJ Glob Heal 2021;6. 10.1136/bmjgh-2020-004564. PMC7992372 33758012

[24] Bin-Ghouth AS, Al-Shoteri S, Mahmoud N, Musani A, Baoom NM, Al-Waleedi AA, et al. SARS-CoV-2 seroprevalence in Aden, Yemen: a population-based study. Int J Infect Dis 2022;115:239–44. doi: 10.1016/j.ijid.2021.12.330 34929358 PMC8677627

