# Correction: High seroprevalence of antibodies against SARS-CoV-2 among healthcare workers 8 months after the first wave in Aden, Yemen

**DOI:** 10.1371/journal.pgph.0002963

**Published:** 2024-02-13

**Authors:** Rami Malaeb, Nagwan Yousef, Omar Al-Nagdah, Qassem Hussein Ali, Mohammed Ali Saleh Saeed, Amna Haider, Evgenia Zelikova, Nada Malou, Sonia Guiramand, Clair Mills, Francisco Luquero, Klaudia Porten

There are errors in the Funding and Competing Interests statements. The correct statements are:

**Funding:** The study was funded by Médecins Sans Frontières (MSF) France. MSF provided support in the form of salaries for NY, OAN, QHA, MASS, EZ, NM, SG, and CM. The specific roles of these authors are articulated in the ‘author contributions’ section. Additionally, two-thirds of Epicentre’s budget is funded by MSF, therefore, MSF indirectly supports Epicentre staff. MSF also provided comprehensive support for this study, including funding for human resources and materials. Specifically, MSF covered the costs of rapid diagnostic tests and supported the Aden National Blood Transfusion and Research Centre’s laboratory work required for our analysis. Coauthors affiliated with MSF contributed to the study design, data collection, and analysis, but the funder otherwise had no role in study design, data collection and analysis, decision to publish, or preparation of the manuscript.

**Competing Interests:** The authors have read the journal’s policy and have the following competing interests to declare: MSF provided support in the form of salaries for NY, OAN, QHA, MASS, EZ, NM, SG, and CM. This does not alter our adherence to *PLOS Global Public Health* policies on sharing data and materials. There are no patents, products in development, or marketed products associated with this research to declare.
