# Supplementary material for: High seroprevalence of antibodies against SARS-CoV-2 among healthcare workers 8 months after the first wave in Aden, Yemen
Source: PLOS Glob Public Health. 2022 Nov 9;2(11):e0000767. doi: 10.1371/journal.pgph.0000767 (PMC10022234; doi:10.1371/journal.pgph.0000767)
Supplement: S1 File — (DOCX) [file pgph.0000767.s001.docx]

**S1 File**

**High Seroprevalence of Antibodies Against SARS-CoV-2 Among Healthcare Workers 8 Months After The First Wave in Aden, Yemen**

Rami Malaeb, Nagwan Yousef, Omar Al-Nagdah, Qassem Hussein Ali, Mohammed Ali Saleh Saeed, Amna Haider, Evgenia Zelikova, Nada Malou, Sonia Guiramand, Clair Mills, Francisco Luquero, Klaudia Porten

**Table A. Seropositivity Results of NG-Test and Elecsys between September 2020 and January 2021 at MSF Aden Trauma centre, Yemen**

|  | Date of screening | number of participants | N  IgG | N  IgM | N  IgG & IgM | Seropositive % | ECLIA reactive  % |
| --- | --- | --- | --- | --- | --- | --- | --- |
| Baseline screening | 27/09/2020 | 320 | 3 | 6 | 43 | 16.25% | - |
| 1st follow-up screening | 11/10/2020 | 230 | 0 | 1 | 13 | 6.08% | - |
| 2nd follow-up screening | 25/10/2020 | 206 | 0 | 1 | 2 | 1.45% | - |
| 3rd follow up screening | 8/11/2020 | 190 | 0 | 0 | 0 | 0% | - |
| final screening | 10/1/2021 | 161 | 1 | 2 | 10 | 8.07% | 67.70% |

**(retest)sultsollow-up cumulative he final round has improv2 and also added tble in supplementary materials s done after / I can**

**Table B. Multivariate analyses for risk factors of seropositivity to SARS-CoV-2 in 161 participants in the MSF Aden Trauma Centre**

|  | **multivariate** | |
| --- | --- | --- |
|  | **AOR CI95%** | **P-value** |
| **Sex** |  |  |
| *Male* | Ref |  |
| *Female* | 1.17 [0.8 - 1.54] | 0.38 |
|  |  |  |
| **Age group** |  |  |
| *20-29* | Ref | - |
| *30-39* | 0.98 [0.6 - 1.4] | 0.92 |
| *40-49* | 1.02 [0.6 - 1.4] | 0.91 |
| *50-59* | 1.5 [0.9 - 2.1] | 0.19 |
| *60+* | 0.5 [-0.09 - 1.09] | 0.07 |
|  |  |  |
| **Type of contract** |  |  |
| *Daily work* | 1.19 [0.8 – 1.6] | 0.4 |
| *Temporary* | 0.75 [0.26 – 1.24] | 0.24 |
| *Permanent* | Ref |  |
|  |  |  |
| **Job role** |  |  |
| *Medical Staff* | Ref | - |
| *Other health personnel* | 0.89 [0.6 - 1.16] | 0.43 |
| *Other personnel at health facility* | 0.43 [0.15 - 0.7] | <0.01* |
|  |  |  |
| **Comorbidities** |  |  |
| *No* | Ref | - |
| *Yes* | 0.8 [0.54 - 1.05] | 0.12 |
|  |  |  |
| **attended IPC Training** |  |  |
| *No* | Ref | - |
| *Yes* | 1.02 [0.8 - 1.2] | 0.8 |
|  |  |  |
| **Contact with suspect/confirmed case in the past 2 weeks** |  |  |
| No | Ref | - |
| yes | 0.72 [0.5 - 0.95] | 0.008* |

**Table C. Univariate and Multivariate analysis risk factors of seropositivity to SARS-CoV-2 in 356 participants in the MSF Aden Trauma Centre (NG-Test results)**

|  | **Univariate** | | **multivariate** | |
| --- | --- | --- | --- | --- |
|  | **OR CI95%** | **P-value** | **AOR CI95%** | **P-value** |
| **Sex** |  |  |  |  |
| *Male* | Ref |  | Ref |  |
| *Female* | 0.47 [0.14 – 0.8] | <0.001 | 0.42 [0.06 – 0.7] | <0.001 |
|  |  |  |  |  |
| **Age group** |  |  |  |  |
| *20-29* | Ref | - | Ref | - |
| *30-39* | 0.88 [0.6 – 1.17] | 0.4 | 0.76 [0.4 – 1] | 0.12 |
| *40-49* | 1.33 [1 – 1.6] | 0.07 | 1.16 [0.8 – 1.5] | 0.4 |
| *50-59* | 1.5 [1.1 – 1.9] | 0.05 | 1.19 [0.7 – 1.6] | 0.4 |
| *60+* | 2.25 [1.7 – 2.8] | 0.004 | 2.06 [1.5 – 2.6] | 0.01 |
|  |  |  |  |  |
| **Type of contract** |  |  |  |  |
| *Daily work* | 1.5 [1.2 – 1.7] | 0.002 | 1.2 [0.9 – 1.5] | 0.2 |
| *Temporary* | 0.3 [-0.2 – 0.8] | <0.001 | 0.4 [-0.09 – 0.9] | <0.001 |
| *Permanent* | Ref |  |  |  |
|  |  |  |  |  |
| **Job role** |  |  |  |  |
| *Medical Staff* | Ref | - | Ref | - |
| *Other health personnel* | 0.4 [0.16 – 0.63] | <0.001 | 0.36 [0.1 – 0.6] | <0.001 |
| *Other personnel at health facility* | 0.46 [0.22 – 0.7] | <0.001 | 0.51 [0.25 – 0.76] | <0.001 |
|  |  |  |  |  |
| **Comorbidities** |  |  |  |  |
| *No* | Ref | - | Ref | - |
| *Yes* | 1.27 [1.05 – 1.5] | 0.03 | 1.25 [1 – 1.5] | 0.07 |
|  |  |  |  |  |
| **attended IPC Training** |  |  |  |  |
| *No* | Ref | - | Ref | - |
| *Yes* | 0.69 [0.5 – 0.87] | <0.001 | 0.7 [0.5 – 0.87] | <0.001 |
|  |  |  |  |  |
| **Contact with suspect/confirmed case in the past 2 weeks** |  |  |  |  |
| No | Ref | - | Ref | - |
| yes | 0.56 [0.3 – 0.7] | <0.001 | 0.5 [0.3 – 0.7] | <0.001 |
